# 

**Published:** 1872-04

**Authors:** 


### CONTENTS.

**COMMUNICATIONS.**

PAGE.

Wholesome Heat for Healthful Homes. B. H. Pratt, A. M. ... 1
Inspectors, Examiners, Censors. Thos. K. Beecher. 3
Education Before Birth. P. H. Hayes, M. D ... 5
Tough, Tougher, Toughest. Dr. Ned ... 6

**CLIPPINGS.**

Colored Deacon's Prayer ... 9
Deaths at Five, A. M ... 9
Promotion of Anatomical Science ... 17

**CORRESPONDENCE.**

PAGE.

Letters on Quackery and Answers to Correspondents...15

**EDITORIAL.**

Medical Items and News ... 9
Domestic Medicine ... 17
To the Point ... 19
The Yardley Swindle ... 19
Felon ... 20
Astigmatism ... 21
Is it Murder ... 22
Eye Cups ... 23
Editorial Chit-Chat ... 25

